# Induction of Bcl-2 Expression by Hepatitis B Virus Pre-S2 Mutant Large Surface Protein Resistance to 5-Fluorouracil Treatment in Huh-7 Cells

**DOI:** 10.1371/journal.pone.0028977

**Published:** 2011-12-22

**Authors:** Jui-Hsiang Hung, Yen-Ni Teng, Lily Hui-Ching Wang, Ih-Jen Su, Clay C. C. Wang, Wenya Huang, Kuan-Han Lee, Kuan-Ying Lu, Lyu-Han Wang

**Affiliations:** 1 Department of Biotechnology, Chia Nan University of Pharmacy and Science, Tainan, Taiwan; 2 Department of Biological Sciences and Technology, National University of Tainan, Tainan, Taiwan; 3 Institute of Molecular and Cellular Biology, National Tsing Hua University, Hsinchu, Taiwan; 4 National Institute of Infectious Diseases and Vaccinology, National Health Research Institutes, Tainan, Taiwan; 5 Department of Pharmacology and Pharmaceutical Sciences, University of Southern California, School of Pharmacy, Los Angeles, California, United States of America; 6 Department of Medical Technology, College of Medicine, National Cheng Kung University, Tainan, Taiwan; 7 Institute of Pharmaceutical Science, Chia Nan University of Pharmacy and Science, Tainan, Taiwan; University of Birmingham, United Kingdom

## Abstract

**Background:**

Hepatocellular carcinoma (HCC) is one of the most common malignancies worldwide with poor prognosis due to resistance to conventional chemotherapy and limited efficacy of radiotherapy. Our previous studies have indicated that expression of Hepatitis B virus pre-S2 large mutant surface antigen (HBV pre-S2Δ) is associated with a significant risk of developing HCC. However, the relationship between HBV pre-S2Δ protein and the resistance of chemotherapeutic drug treatment is still unclear.

**Methodology/Principal Findings:**

Here, we show that the expression of HBV pre-S2Δ mutant surface protein in Huh-7 cell significantly promoted cell growth and colony formation. Furthermore, HBV pre-S2Δ protein increased both mRNA (2.7±0.5-fold vs. vehicle, *p* = 0.05) and protein (3.2±0.3-fold vs. vehicle, *p* = 0.01) levels of Bcl-2 in Huh-7 cells. HBV pre-S2Δ protein also enhances Bcl-2 family, Bcl-xL and Mcl-1, expression in Huh-7 cells. Meanwhile, induction of NF-κB p65, ERK, and Akt phosphorylation, and GRP78 expression, an unfolded protein response chaperone, were observed in HBV pre-S2Δ and HBV pre-S-expressing cells. Induction of Bcl-2 expression by HBV pre-S2Δ protein resulted in resistance to 5-fluorouracil treatment in colony formation, caspase-3 assay, and cell apoptosis, and can enhance cell death by co-incubation with Bcl-2 inhibitor. Similarly, transgenic mice showed higher expression of Bcl-2 in liver tissue expressing HBV pre-S2Δ large surface protein *in vivo*.

**Conclusion/Significance:**

Our result demonstrates that HBV pre-S2Δ increased Bcl-2 expression which plays an important role in resistance to 5-fluorouracil-caused cell death. Therefore, these data provide an important chemotherapeutic strategy in HBV pre-S2Δ-associated tumor.

## Introduction

Hepatocellular carcinoma (HCC) is a common malignancy affecting approximately one million people worldwide annually [1]. It is one of the most common causes of cancer morbidity and mortality in Asia and Africa. Hepatocellular neoplasms develop regularly from preneoplastic foci of altered hepatocytes, and hepatocellular cancer occurs both sporadically and in relation to chronic viral infection [2], environmental exposure [3], extensive alcohol intake [4], transgenic oncogenes [5], [6], [7] and alternative causes of hepatic cirrhosis. HBV is considered a major etiological factor in the development of HCC. Chronic HBV carriers have a greater than 100 fold increased relative risk of developing HCC, although the oncogenic mechanisms of HBV are not completely understood [8], [9]. HBV encodes three envelope proteins in the pre-S/S open reading frame that are named large, middle, and small surface proteins. The expression of HBV surface proteins is related to liver tumor development and a number of truncated surface gene mutants with a partially deleted pre-S region were already identified [10], [11], [12], [13], [14]. One of the major mutant types is the deletion of the pre-S2 region (pre-S2Δ). These pre-S2Δ mutants are becoming increasingly prevalent in serum and liver tissues of patients with chronic HBV infection and HCC [15], [16], [17].

HBV surface mutant pre-S2Δ gene is deleted in approximately nucleotides 2 to 55 of the pre-S_2_ region and often contains a point mutation in the start codon of the pre-S2 region. The pre-S2Δ type of HBV large surface mutant protein is predominant in hepatocellular carcinoma patients with HBV infection [18], [19]. Based on epidemiologic studies, HBV carriers who presented with the pre-S2Δ mutant protein in serum had worse disease outcomes than those who did not [20]. Overexpression of pre-S2Δ large surface proteins has been demonstrated in the induction of endoplasmic reticulum (ER) stress [21]. Unfolded proteins in the endoplasmic reticulum activate several signaling pathways that are referred to as the unfolded protein responses (UPR). UPR pathway has three components in mammalian cells: basic leucine zipper transcription factor ATF6, IRE1 RNA-processing enzyme, and ER localized kinase (PERK). Previous studies have indicated that activation of NF-κB is through calcium release, reactive oxygen species production, IRE1, and PERK signal pathway during ER stress [22], [23], [24], [25], [26]. We have also characterized the NF-κB response and found that NF-κB was activated through multiple pathways, including calcium signaling and pp38 kinase [21]. Activation of NF-κB by ER stress leads to induction of many cellular genes that are largely anti-apoptosis in function.

HCC is one of the most common malignancies worldwide with poor prognosis due to resistance to conventional chemotherapy and limited efficacy of radiotherapy. A major challenge in the systemic treatment of HCC is cellular resistance to conventional cytotoxic agents. In addition, many studies have indicated that alternation of gene expression is correlated with drug resistance, such as overexpression of protein kinase C, EGF receptor, c-erbB2, c-ras and c-Bcl-2 in tumor cells [27], [28], [29], [30]. Previous studies have indicated a high-level expression of Bcl-2 in many tumor tissues, including HCC [31], [32]. The *Bcl-2* gene family is a group of apoptosis-related genes which have been studied extensively. Bcl-2 protein overexpression is associated with drug resistance and poor clinical outcome in cancer patients. Apoptosis is mainly executed by proteases of caspase family, whereby members of Bcl-2 family, such as Bcl-2, Bcl-xL, or Mcl-1, inhibit caspase activation [33], [34]. The proteins of Bcl-2 family are important regulators of apoptosis induced by a wide array of stimuli, including chemotherapeutic drugs.

From our previous study, HBV pre-S2Δ large surface protein expression in hepatocytes has been correlated with hepatocarcinogenesis. For patients with cancer who are from HBV-endemic areas, routine screening for HBsAg before cytotoxic chemotherapy should be performed, and there was a trend of poorer survival for patients who had developed severe hepatitis during chemotherapy [35]. Furthermore, a recent study indicated that a pre-chemotherapy for high HBV viral levels is associated with poorer survival in HCC patients with chronic HBV infection [36], and the occurrences of HBV pre-S2Δ large surface proteins in HBV cancer patients are about 30% in Taiwan [18]. On the other hand, previous study has also indicated Bcl-2 protein expression in acute and chronic hepatitis, cirrhosis and hepatocellular carcinoma [37]. However, the relationship between HBV pre-S2Δ large surface protein and Bcl-2 expression or drug resistance is still unclear. Therefore, in this study, we employed HBV pre-S2Δ large surface protein expressing cells to analyze altered expressed proteins in hepatoma Huh-7 and immortalized hepatocyte NeHepLxHT cell lines. We identified induction of Bcl-2 family overexpression by HBV pre-S2Δ large surface protein in Huh-7 cells. Furthermore, HBV pre-S2Δ large surface protein expressing cell was more resistant to 5-fluorouracil treatment. However, the ability of 5-fluorouracil resistance was significantly decreased by co-incubating 5-fluorouracil with Bcl-2 inhibitor. We found that HBV large surface protein and HBV pre-S2Δ large surface protein enhanced Bcl-2 family expression and resistance to chemotherapeutic 5-fluorouracil treatment. Taken together, these results suggest that co-treatment of Bcl-2 inhibitor might be important for chemotherapeutic strategy of HBV pre-S2Δ large surface protein containing tumor cells.

## Materials and Methods

### Cell culture and chemicals

Huh-7 and cell line was obtained from ATCC (Manasses, VA, USA). Huh-7 pre-S2Δ and Huh-7 pre-S cell lines were generated from Huh-7. NeHepLxHT and NeHepLxHT pre-S2Δ cell lines were gift from Dr. Lily Hui-Ching Wang. NeHepLxHT cell line was derived by exogenous expression of human telomerase reverse transcriptase (hTERT) in normal human neonatal hepatocytes. These cell lines were maintained at 37°C in a 5% CO_2_ atmosphere in DMEM supplemented with 10% heat-inactivated fetal bovine serum, 100 units/ml penicillin, and 100 µg/ml streptomycin (Invitrogen, Ground Island, NY, USA). Culture medium was replaced every 2 days. ECL Western blot detection system was purchased from Millipore (Billerica, MA, USA). Anti-GRP78 was purchased from Transduction Laboratories. Anti-Bcl-2, anti-Bcl-xL, anti-Mcl-1, anti-Bax, anti-Bak, anti-p-Ser^311^-p65, anti-p65, anti-MCL-1 anti-Bcl-xL, anti-Bax, anti-Bak, anti-ERK, anti-phospho-p44/42 MAPK, Akt, and p-Thr^308^-Akt antibodies were obtained from Santa Cruz Biotechnology (Santa Cruz, CA, USA). Anti-p-Ser^276^-p65, anti-rabbit IgG-horseradish peroxidase (HRP) conjugates, and rabbit anti-mouse IgG-HRP conjugates antibodies were purchased from Cell Signaling (Beverly, MA, USA). Anti-HA antibody and Bcl-2 inhibitor II, YC137 were purchased from Calbiochem (San Diego, CA, USA).

### Plasmid and Stable Clone Cell Lines Construction

Plasmid pIRES, pIRES-preS-HA, and pIRES-pre-S2Δ-HA were gifts from Dr. Wenya Huang. Huh-7 cells were transfected with pIRES, pIRES-preS-HA, and pIRES-pre-S2Δ-HA plasmids using Invitrogen LipofectAMINE 2000 reagent according to the manufacturer's protocol. Cells were then selected by G418 for 2 weeks. The expression of HBV large surface proteins in pIRES-pre-S and pIRES-pre-S2Δ stable clone cell line were confirmed by Western blotting.

### Western blot analysis

The cell lysates were collected with RIPA lysis buffer (50 mM Tris-cl pH 7.4. 150 mM NaCl. 1% NP40. 0.25% Na-deoxycholate. 1 mM PMSF, 1 mM EDTA; 5 µg/ml Aprotinin.) containing protease inhibitors (1 mM PMSF, 1 mM orthovanadate, 1 mM EDTA, and 10 µg/mL: leupeptin). Protein concentrations of cell lysates were measured using a Micro BCA protein assay reagent kit (Pierce, Rockford, IL, USA). To the cell lysate, the same volume of SDS-PAGE sample loading buffer [100 mmol/L Tris-HCl, 4% SDS, 5% β-mercaptoethanol, 20% glycerol, and 0.1% bromphenol blue (pH 6.8)] was added, and the cell lysates were boiled for 10 min. Equal amounts of proteins were resolved in 10 or 12% SDS-polyacrylamide gels and transferred to nitrocellulose membranes using a semidry transfer cell. The blotted membrane was washed twice with TBS containing 0.1% Tween 20 (TBST; 10 mM Tris-HCl, pH 7.5, 150 mM NaCl, 0.05% Tween-20). After blocking with TBST containing 5% nonfat milk for 1 h, the membranes were probed with the following antibodies against: HA, β-actin (43 Kda), GRP78 (78 kDa), Bcl-2 (26 kDa), p65 (65 kDa), p-Ser276-p65 (65 kDa), p-Ser311-p65 (65 kDa), Bcl-xL (30 kDa)), MCL-1 (40 kDa), Bax (23 kDa), Bak (30 kDa), Akt (62 kDa), pAkt (62 kDa), ERK (44 kDa), and pERK (44 kDa) antibodies in 1% TBST nonfat milk at 4°C overnight. The membrane was washed thrice with TBST for a total of 15 min. The secondary anti-mouse IgG-HRP conjugates or anti-rabbit IgG-HRP conjugates (1∶2,000 dilutions) was subsequently incubated with the membrane for 1 h at room temperature and was washed extensively for 50 min with TBST. The blots were visualized with the enhanced chemiluminescence (GE, Pittsburgh, PA, USA), and according to the manufacturer's instructions. The blots were developed with the ECL-Western blot detection system according to the manufacturer's instruction.

### Cell viability

Cell viability was assessed using the trypan blue staining assay in three replicates. Huh-7 and stable transfectant cells were seeded at 1×10^4^ per well in 24-well flat-bottomed plates and incubated in 10% FBS–supplemented DMEM for 24 h. Cells were treated with 0.1, 0.25, and 0.5 mM 5-fluorouracil in the same medium. Controls received the DMSO vehicle at a concentration same as that in drug-treated cells. After 4 days, cell viability was determined by trypan blue exclusion and microscopy examination.

### Semi-quantitative RT-PCR

The cells were washed with cold PBS and then harvested. Total RNA was extracted using Trizol Reagent (Invitrogen, Ground Island, NY, USA). The cDNA was reverse-transcribed from 1 mg of total RNA using oligo(dT) primers and Moloney murine leukemia-virus transcriptase. The following primer sequences were employed to detect Bcl-2 transcripts: sense (5′-GCACCCACTCCCTTCATACAAT-3′) and antisense (5′-ACGCAGGTTACATTCGTCTTCC-3′. PCR reaction was performed as follows: reverse transcription at 42°C for 60 min and denaturation at 72°C for 2 min; then amplification for 30 cycles at 94°C for 30 s, annealing at 56°C for 30 s, and extension at 72°C for 30 s, followed by a terminal elongation step at 72°C for 10 min and a final holding stage at 4°C). Meanwhile, the same amount of cDNA was amplified for 25 cycles using specific glyceraldehyde-3-phosphate dehydrogenase primers: 5′-TGAAGGTCGGTGTGAACGGATTTGGC-3′ (sense) and 5′-CATGTAGGCCATGAGGTCCACCAC-3′ (antisense). The products were visualized after electrophoresis on a 1.5% agarose gel containing ethidium bromide. The signal level of the bands was quantified densitometrically.

### Colony formation assay

For colony formation, Huh-7 and stable transfectant cells were seeded at 1000 per well in 6-well flat-bottomed plates and incubated in 10% FBS–supplemented DMEM for 24 h. The cells were treated with 5-fluorouracil as dose indicated and 0.25 mM 5-fluorouracil plus 1 or 5 µM Bcl-2 inhibitor (Bcl-2 inhibitor II, YC137) for 24 h. The culture medium was replenished, and cells were maintained at 37°C for 14 days with medium changed every other day. Grown colonies were fixed with 3.7% formaldehyde and stained with crystal violet. The number of cell colonies was determined directly on each well.

### Analysis of caspase-3 activity

Caspase-3 activity was determined using PE active caspase-3 apoptosis kit (BD Pharmingen). Briefly, Huh7 V, pre-S, and pre-S2Δ (5×10^5^) cells in 10-cm dishes were subjected to different drug treatments as indicated for 72 h and were resuspended cells in 0.5 ml Cytofix/Cytoperm solution for 20 min on ice. Furthermore, cells were incubates in 100 µl of Perm/Wash buffer containing 20 µl caspase-3 antibody for 30 min at room temperature. Each sample was then added with 400 µl Perm/Wash buffer, and caspase-3 activity signals were analyzed by flow cytometry.

### Annexin V/propidium iodide assay

For assessment of apoptosis, both floating and adherent cells were collected and analyzed. Briefly, 5×10^5^ cells per dish were plated onto 6-cm dishes and incubated at 37°C for 16 h. The cells were treated with 5-fluorouracil alone or plus Bcl-2 inhibitor for 24 h. The cells were washed twice with PBS and collected by trypsinization. After centrifugation at 400× g for 5 min at room temperature, the cells were stained with Annexin V and propidium iodide (1 µg/mL). The cell apoptosis distributions were determined on a FACScort flow cytometer and analyzed by ModFitLT V3.0 software program.

### Transgenic Mice Tissue Protein Extraction

The transgenic mouse liver tissues were gifts from Dr. Ih-Jen Su. The pre-S2Δ transgenic mice were constructed by injection of pre-S2Δ gene fragment into the male pronucleus of fertilized mouse ova. Microinjection was performed in Fvb/n mice. After 12 months, liver tissue from pre-S2Δ transgenic mice was homogenized in RIPA buffer (50 mM Tris-HCl (pH 7.4), 1% Nonidet P-40, 0.25% sodium deoxycholate, 150 mM NaCl, 1 mM EDTA, 1 mM phenylmethylsulfonyl fluoride, 1 µg/ml aprotinin, leupeptin, and pepstatin, 1 mM Na3VO4, and 1 mM NaF). Homogenates were centrifuged at 15,000× g for 10 min at 4°C, and the supernatants were collected. Total protein concentrations of the tissue lysates were quantified using the Micro BCATM protein assay reagent kit following the manufacturer's instructions.

### Immunohistochemical Staining

Immunohistochemical staining was performed on depar-affinized tissue sections of formalin-fixed material. For immunohistochemical staining, 4-mm-thick paraffin sections were stained with mouse anti-preS or anti-Bcl-2. The primary antibodies and working dilutions were as follows: HBsAg (1∶500, 7H11; gift from Dr. Wenya Huang), Bcl-2 (1∶100, polyclonal N-19; Santa Cruz Biotechnology, Santa Cruz, CA). Detection was done with streptavidin-biotinylated peroxidase-conjugated reagents (LSAB+ kit; DAKO, Carpinteria, CA). A biotinylated anti-mouse secondary antibody (DAKO) was then applied and incubated with peroxidase-conjugated streptoavidin, chromogenized by 3-amino-9-ethylcarbazole.

### Statistical Analysis

Results were presented as the mean ± S.D., and statistical comparisons were made using the Student's *t* test. Significance was defined at the *p*<0.05 or 0.01 levels.

## Results

### Expression of pre-S and pre-S2Δ proteins in Huh-7 cell

**Figure 1:**
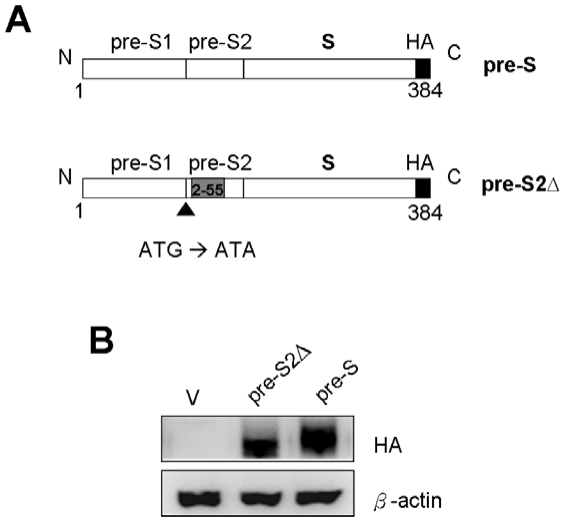
Expression of HBV pre-S2Δ large surface protein in Huh-7 cells. Schematic representation of various lengths of HBV surface protein. (A) The pre-S represents full length (384 a.a). S represents C-terminal of pre-S2Δ (158–384 a.a.) Numbers in each construct correspond to amino acid positions. The arrow represents the point mutation of pre-S2 start codon from ATG to ATA. Gray box indicates pre-S2 deletion region. (B) Huh-7 cells were transfected with pIRES-pre-S2Δ-HA (pre-S2Δ), pIRES-pre-S-HA (pre-S), or pIRES vector control (V). The expression of pre-S2Δ protein and pre-S in Huh-7 cells were analyzed by Western blotting with antibody for HA. β-actin was used as a loading control.

To establish the expression of HBV pre-S large surface protein and HBV pre-S2Δ large surface protein in hepatoma Huh-7 cell line. HBV large surface wild type HBS gene and mutant HBS gene were constructed into pIRES plasmid (Fig. 1A). Huh-7 cells were transfected with pIRES, pIRES-pre-S-HA, and pIRES-pre-S2Δ-HA plasmids, and the transfectants were selected with G418. The expression of HBV large surface proteins in pIRES-pre-S2Δ-HA and pIRES-pre-S-HA stable clone cell lines were confirmed by Western blotting. The result shows that HBV pre-S large surface protein and HBV pre-S2Δ large surface protein can be expressed in Huh-7 cells (Fig. 1B).

### The pre-S and pre-S2Δ large surface proteins enhance cell growth in Huh-7 cell line

**Figure 2:**
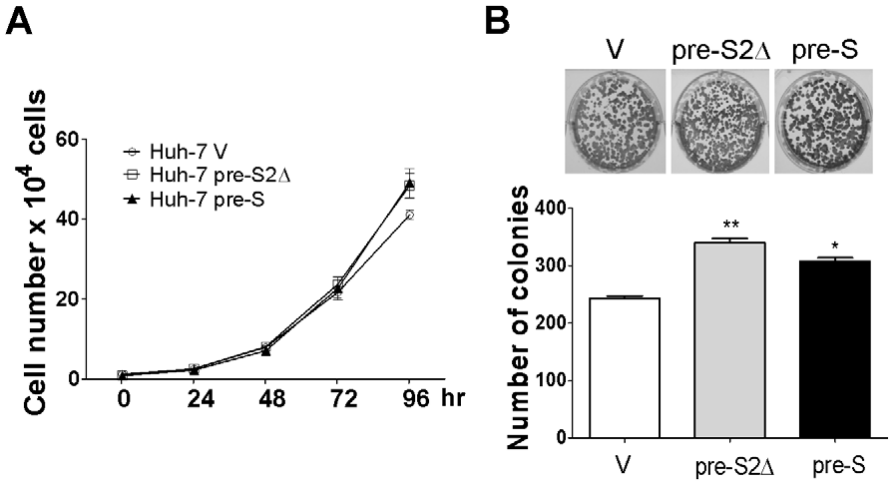
HBV pre-S2Δ large surface protein increases cell growth and colony formation. (A) The effect of HBV large surface protein on Huh-7 growth rate was determined and cells were maintained in FBS–supplemented DMEM for 4 days, and the number of cells was assessed by trypan blue staining assay. (B) Huh-7 stable cell lines were analyzed for colony formation ability by colony formation assay, and colony formation was scored after 7 days. The number of colonies in the graphs was representative of three independent experiments (lower panel). Data represent the mean ± SD (n = 3). Significant differences (*, *P*<0.05 and **, *P*<0.01) between the control and experimental group are marked with an asterisks.

The characteristics of pre-S and pre-S2Δ large surface proteins in Huh-7 cells are investigated for cell growth rate. The stable cell lines of pre-S and pre-S2Δ large surface proteins were confirmed using trypan blue staining assay and colony formation assay. The result indicated that the growth rate of pre-S and pre-S2Δ stable cell lines increased at 72–96 h (Fig. 2A). Furthermore, in order to eliminate the possibility that pre-S and pre-S2Δ large surface proteins affect the colony-forming ability in Huh-7 cell line, we determined the number colonies in Huh-7 cells cultured for 14 days. As shown in Fig. 2B, Huh-7 pre-S2Δ cells produced the largest number of colonies, 399±7. Huh-7 pre-S cells produced fewer colonies, 307±6, and Huh-7 V cells produced the smallest number of colonies, 242±5 (Fig. 2B).

### Induction of Bcl-2 family expression in Huh-7 pre-S2Δ large surface protein cell line

**Figure 3:**
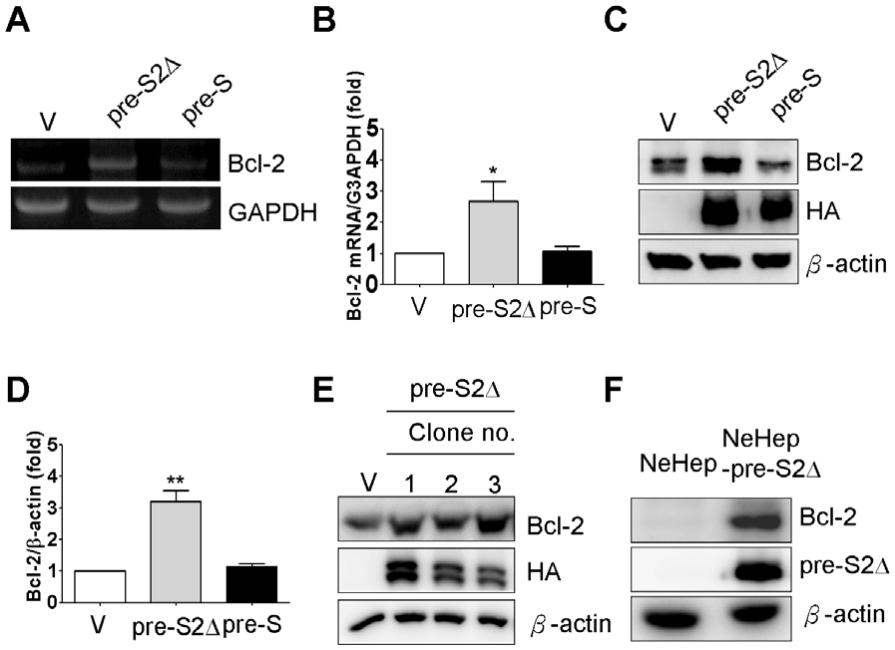
Elevated Bcl-2 expression in HBV pre-S2Δ cells. (A) Total RNA was isolated from Huh-7 -V, Huh-7 pre-S2Δ, and Huh-7 pre-S cells and then subjected to RT-PCR analysis with Bcl-2 and G3APDH specific primers. (B) For Huh-7 -V, Huh-7 pre-S2Δ, and Huh-7 pre-S cells, the relative levels of Bcl-2 mRNA were quantified. The data represent the mean of Bcl-2 mRNA expression level from three independent experiments. Columns, mean; bars, SD (n = 3). Significant differences (**, *P*<0.01) between the control and experimental group are marked with an asterisks. (C) The cell lysates collected from three stable clones, and cell lysates were analyzed by Western blotting analysis with antibodies for hemagglutinin (HA) tag, Bcl-2, and β-actin. (D) The levels of Bcl-2 protein in the graphs were representative of three independent experiments (lower panel). Columns, mean; bars, SD (n = 3). Significant differences (**, *P*<0.01) between the control and experimental group are marked with an asterisks. (E) Furthermore, expression level of Bcl-2 was determined by using Western blotting in three individual pre-S2Δ stable cell lines. (F) Expression level of Bcl-2 was enhanced by pre-S2Δ in the immortalized human hepatocyte cell line. Total cell lysate from NeHep and NeHep-pre-S2Δ were determined by Western blotting analysis with Bcl-2, pre-S, and β-actin specific antibosies.

Many studies have indicated that overexpression of Bcl-2 family was correlated with chemotherapeutic drug resistance. To observe the effect of pre-S2Δ and pre-S large surface proteins on Bcl-2 family expression in Huh-7 cells, the expression of Bcl-2 was determined by RT-PCR and Western blotting assay for Huh-7 V, Huh-7 pre-S2Δ, and Huh-7 pre-S cell line with specific Bcl-2 primer and Bcl-2 antibody. First, the total RNA was extracted from three Huh-7 stable cell lines, and RT-PCR to quantify the Bcl-2 mRNA. High expression of Bcl-2 mRNA was observed in hepatoma cells that express the deletion forms of HBV large surface proteins (Fig. 3A and Fig. 3B). Furthermore, the result shows that the expression levels of Bcl-2 protein were significantly increased in Huh-7 pre-S2Δ cell. For example, expression level of Bcl-2 in Huh-7 pre-S2Δ cell was enhanced to about 3-fold compared with that in Huh-7 V cell (Fig. 3C and Fig. 3D). To reconfirm the induction of Bcl-2 expression by pre-S2Δ proteins, we analyzed three additional clones of pre-S2Δ stable cell lines for the levels of Bcl-2 expression. The result shown that increased of Bcl-2 protein expression was also observed in those three additional preS-S2Δ stable cell lines (Fig. 3E). Furthermore, to evaluate the event of induction of Bcl-2 expression by pre-S2Δ proteins manner in human hepatocyte, Bcl-2 expression level was determined in NeHepLxHT and NeHepLxHT pre-S2Δ cell lines. The result indicated that expression of Bcl-2 was increased by pre-S2Δ proteins in human hepatocyte NeHepLxHT cells (Fig. 3F).

### Increased Bcl-2 family expression in Huh-7 pre-S2Δ large surface protein cell line

**Figure 4:**
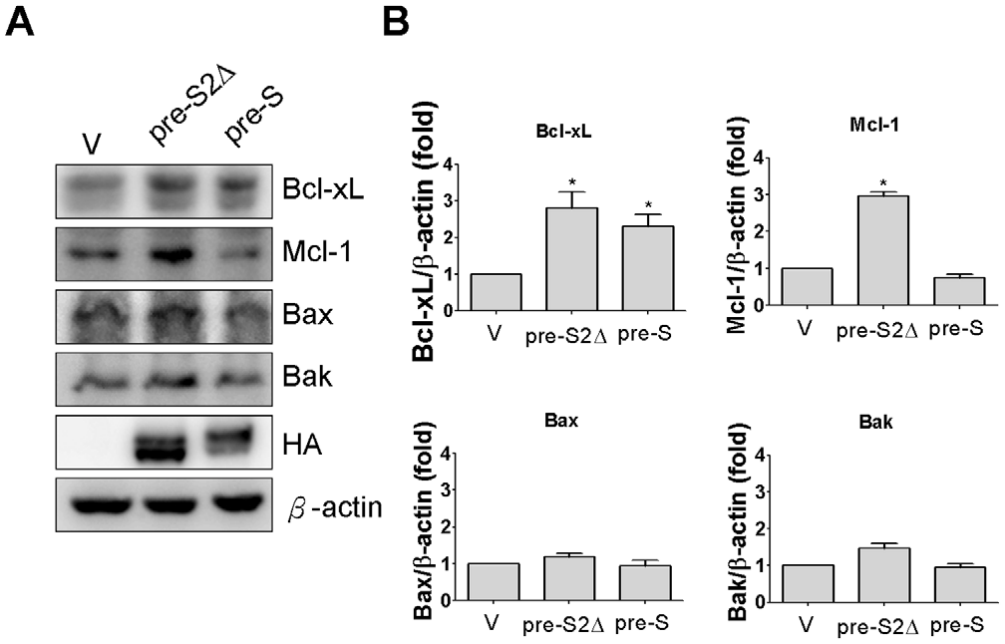
The pre-S2Δ large surface protein induced expression of Bcl-2 family proteins. (A) Total cell lysate was isolated from Huh-7 -V, Huh-7 pre-S2Δ, and Huh-7 pre-S cells and then subjected to Western blotting analysis with Bcl-xL, Mcl-1, Bax, and Bad specific antibodies. (B) For Huh-7 -V, Huh-7 pre-S2Δ, and Huh-7 pre-S cells, the relative levels of Bcl-xL, Mcl-1, Bax, and Bad protein were quantified. The data represent the mean of Bcl-xL, Mcl-1, Bax, and Bad expression level from three independent experiments. Columns, mean; bars, SD (n = 3). Significant differences (*, *P*<0.05) between the control and experimental group are marked with an asterisks.

Induction of Bcl-2 expression by pre-S2Δ proteins was observed in Huh-7 cells. Furthermore, expression of Bcl-2 family was also estimated in Huh-7 V, pre-S2Δ, and pre-S cell lines, and the data indicated that Bcl-2 family, Bcl-xL and MCL-1, were increased in Huh-7 pre-S2Δ cells (Fig. 4A). For example, the Bc-xL and MCL-1 expression levels in Huh-7 pre-S2Δ cells were enhanced to about 2.5 and 3-fold compared with Huh-7 V cells (Fig. 4B). Notably, Bcl-xL expression levels were also induced to about 2-fold in Huh-7 pre-S cells.

### Induction of NF-κB, Akt and ERK phosphorylation by HBV pre-S2Δ and pre-S large surface protein

**Figure 5:**
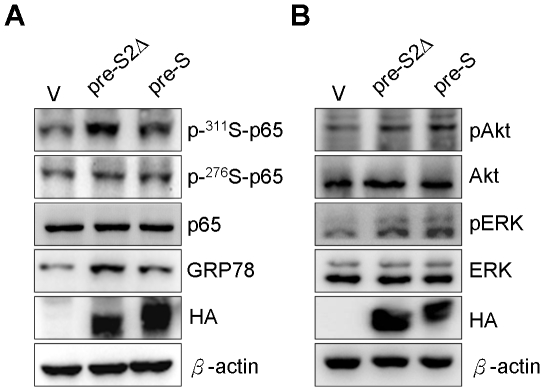
Expression of pre-S2Δ large surface protein increased ER stress and NF-κB, ERK, and Akt phosphorylation in Huh-7 cells. (A) (B) Cells were maintained in FBS–supplemented DMEM and cell lysates were obtained by RIPA lysis buffer. The cell lysates were determined by Western blotting using antibodies specific for GRP78, NF-κB p65, p-Ser276 p65, p-Ser311 p65, ERK, p-ERK, Akt, p-AKTand β-actin.

Previous studies have indicated that expression of viral protein can cause ER stress and NF-κB activation [21], [38], [39]. Our previous results also demonstrated that ER stress induced NF-κB activation and nuclear localization. Therefore, to investigate the effect of pre-S2Δ and pre-S large surface proteins on ER stress and NF-κB activation in Huh-7 cells, the expression of GRP78 and NF-κB phosphorylation were analyzed by Western blotting. The expression of GRP78, an unfolded protein response chaperone, was enhanced by HBV pre-S2Δ and pre-S large surface proteins. Phosphorylation of NF-κB at ser276 and ser311 sites was increased in HBV pre-S2Δ and pre-S-expressing cells (Fig. 5A). In addition, the ERK MAP kinase and PI3-kinase/Akt pathways are major intracellular signaling modules, which are known to regulate diverse cellular processes including cell proliferation, survival and malignant transformation. The ERK and Akt phosphorylation status were determined by Western blotting. The result shows that induction of ERK and Akt phosphorylation were observed in Huh-7 pre-S2Δ and pre-S cell lines (Fig. 5B).

### Differential susceptibility of Huh-7 V, Huh-7 pre-S2Δ, and Huh-7 pre-S cell lines to 5-fluorouracil-induced cell death

**Figure 6:**
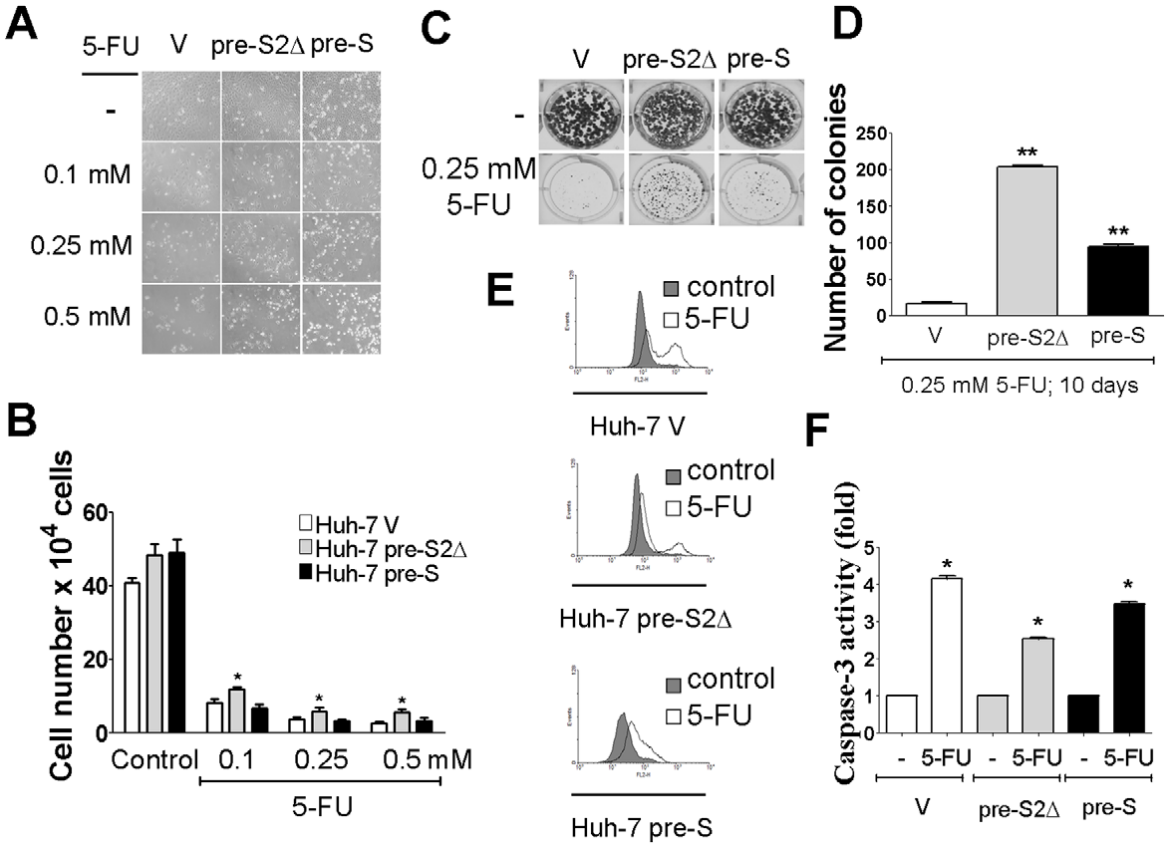
Expression of pre-S2Δ contributes to 5-fluorouracil treatment in Huh-7 cells. (A) The morphologic changes after a 96-hour 0.1 mM, 0.25 mM and 0.5 mM 5-fluorouracil treatment of Huh-7 V, Huh-7 pre-S2Δ, and Huh-7 Pre-S cells. The cells were followed by photography under phase-contrast magnification. (B) Cytotoxicity of 5-fluorouracil to Huh-7 V, Huh-7 pre-S2Δ and Huh-7 pre-S cells were analyzed by trypan blue staining assay. *Columns*, mean of three independent experiments; bars, SD (n = 3). Significant differences (*, *P*<0.05) between the control and experimental group are marked with an asterisks (C) Cell viability after treatment with 5-FU. Huh-7 V, Huh-7 pre-S2Δ and Huh-7 pre-S cells (1×10^3^) were seeded in 6-well plate, as described in Materials and Methods. Cells were then incubated for another 10 days, and the colony formation ability of three cell lines were evaluated using a crystal violet assay determined by crystal violet in response to 5-fluorouracil. (D) A quantitative measure of colony formation was determined by crystal violet and the number of colonies in the graphs was representative of three independent experiments (lower panel). Columns, mean; bars, SD (n = 3). Significant differences (**, *P*<0.01 and ***, *P*<0.001) between the control and experimental group are marked with an asterisks. (E) Flow cytometric analysis the effect of 5-fluorouracil on increasing caspase-3 activity in Huh7 V, Huh-7 pre-S2Δ, and Huh-7 pre-S cells, increased caspase-3 activity was observed in Huh7 V, Huh-7 pre-S2Δ, and Huh-7 pre-S cells, after 72-h exposure to 5-Fluorouracil. (F) The relative caspase-3 activities, normalized to DMSO control, at the indicated 0.25 mM of 5-fluorouracil. *Columns*, mean of three independent experiments; bars, SD (n = 3). Significant differences (*, *P*<0.05) between the control and experimental group are marked with an asterisks.

The in vitro antitumor efficacy of 5-fluorouracil in three human HCC stable cell lines, Huh-7 V, Huh-7 pre-S2Δ, and Huh-7 pre-S were evaluated. As these three stable cell lines harbor vector only and different HBV large surface proteins, these stable cell lines were treated with 5-fluorouracil at different doses indicated. To investigate whether HBV pre-S2Δ large protein was involved in cytotoxic drug resistance mechanisms, the cell viability was determined by trypan blue staining assay and colony formation. As shown in Figure 5A, the 72-hour 5-fluorouracil treatment resulted in dose-dependent, progressive morphological changes from flat to round in Huh-7 V and Huh-7 pre-S cells. In contrast, only minor morphologic changes in Huh-7 pre-S2Δ cells were observed after 72-hours 5-fluorouracil treatment (Fig. 6A). They showed differential susceptibility to the antiproliferative effect of 5-fluorouracil. Huh-7 pre-S2Δ cells exhibited better cell survival compared with Huh-7 V or Huh-7 pre-S (Fig. 6B). Furthermore, the effect of 5-fluorouracil on these three cell lines was evaluated by colony formation assay (Fig. 6C). The number of colonies was quantified from violet staining and Huh-7 pre-S2Δ exhibited largest number of colonies after 5-fluorouracil treatment (Fig. 6D). It is noteworthy that Huh-7 pre-S cell produced more colonies than Huh-7 V cell. To assess the effect of 5-fluorouracil on cell apoptosis, caspase-3 activity was also determined. Flow cytometric analysis of caspase-3 activity showed that 5-fluorouracil treatment was significantly increased caspase-3 activity in Huh-7 V and Huh-7 pre-S cells and, to a lesser extent, Huh-7 pre-S2Δ cells (Fig. 6E). For example, 5-fluorouracil caused 4.2-fold and 3.5-fold increases in caspase-3 activity at 0.25 mM treatment for Huh-7 V and Huh-7 pre-S cell lines. However, caspase-3 activity in Huh-7 pre-S2Δ cells, there is only 2.5-fold increased after 5-fluorouracil treatment (Fig. 6F).

### Bcl-2 inhibitor decreased colony formation and increased caspase-3 activity in Huh-7 pre-S2Δ cells under 5-fluorouracil treatment

**Figure 7:**
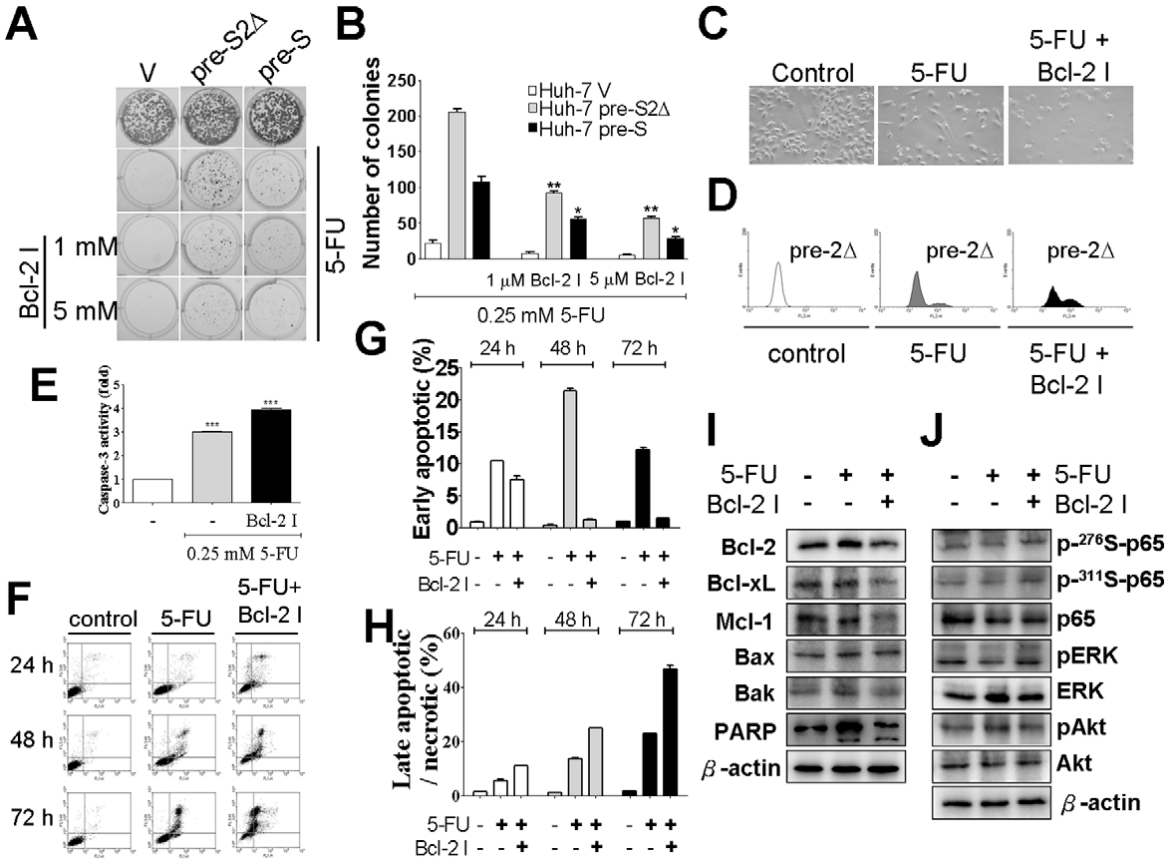
Expression of Bcl-2 contributes to 5-fluorouracil resistance in Huh-7 pre-S2Δ cells. (A) Huh-7 V, Huh-7 pre-S2Δ and Huh-7 pre-S cells (1×10^3^) were seeded in 6-well plates. Cells were treated with 5-fluorouracil with or without Bcl-2 inhibitor, as described in Materials and Methods, and then incubated for another 10 days, and the colony formation ability of three cell lines were evaluated using a crystal violet assay determined by crystal violet in response to 5-fluorouracil. (B) A quantitative measure of colony formation was determined by crystal violet and the number of colonies in the graphs was representative of three independent experiments (lower panel). Data represent the mean ± SD (n = 3). Significant differences (**, *P*<0.01 and ***, *P*<0.001) between the control and experimental group are marked with an asterisks. (C) The morphologic changes after a 72-hour 0.25 mM 5-fluorouracil with or without Bcl-2 inhibitor treatment of Huh-7 pre-S2Δ cells. (D) The effect of 5-fluorouracil or combination with Bcl-2 inhibitor on caspase-3 activity in Huh-7 pre-S2Δ cell line. Enzymatic activity of caspase-3 was determined by caspase-3 antibody, as described under “materials and methods”. (E) The relative caspase-3 activities, normalized to DMSO control, at the indicated 0.25 mM 5-fluorouracil or with 5 µM Bcl-2 inhibitor. *Columns*, mean of three independent experiments; bars, SD (n = 3). Significant differences (***, *P*<0.001) between the control and experimental group are marked with an asterisks. (F) Both apoptosis and necrosis were involved in 5-Fluorouracil or combination with Bcl-2 inhibitor-induced cell death. Huh-7 pre-S2Δ cells were treated with 5-fluorourail with or without Bcl-2 inhibitor and cells were analyzed by annexin V assay. (G) (H) The ratio of apoptotic and necrotic cells, normalized to DMSO control, at the indicated 0.25 mM 5-fluorouracil or with 5 µM Bcl-2 inhibitor. *Columns*, mean of three independent experiments; bars, SD (n = 3). Significant differences (*, *P*<0.05; ***, *P*<0.001) between the control and experimental group are marked with an asterisks. (I) Downregulation of Bcl-2 family expression by combination 5-fluorouracil with Bcl-2 inhibitor. Cells were treated with 5-fluorourail with or without Bcl-2 inhibitor and cell lysates were obtained by RIPA lysis buffer. The cell lysates were determined by Western blotting using antibodies specific for Bcl-2, Bcl-xL, Mcl-1, Bax, Bak and β-actin. (J) The effect of 5-fluorouracil or combination with Bcl-2 inhibitor on Akt, ERK and NF-κB phosphorylation in Huh-7 pre-S2Δ cell line. The total lysates were analyzed by Western blotting using ERK, p-ERK, Akt, p-Akt, p65, p-ser^276^-p65, p-ser^311^-p65 and β-actin antibodies.

We observed that induction of Bcl-2 expression was enhanced by HBV pre-S2Δ large protein in Huh-7 cells, and the cells were resistant to 5-fluorouracil treatment. To determine whether the induction Bcl-2 expression by HBV pre-S2Δ large protein was corrected with resistant to 5-fluorouracil in Huh-7 cells, the combined effect of Bcl-2 inhibitor (Bcl-2 Inhibitor II, YC137) and 5-fluorouracil was evaluated by colony formation assay, caspase-3 activity assay. First of all, cells were exposed to 5-fluorouracil agent or a combination of 5-fluorouracil with Bcl-2 inhibitor II, YC137. After incubation, the number of colonies was determined by staining with crystal violet (Fig. 7A). The data show us that when co-incubated 0.25 mM 5-fluorouracil with 1 µM or 5 µM Bcl-2 inhibitor, it significantly decreased colony formation ability in Huh-7 pre-S2Δ and Huh-7 pre-S cells (Fig. 7B). However, in Huh-7 pre-S2Δ cell line, the colony formation ability was more dramatically decreased by co-treatment 5-fluorouracil with Bcl-2 inhibitor II Y137. In addition, we also observed changes in Huh-7 pre-S2Δ cell morphology after 5-fluorouracil with or without Bcl-2 inhibitor treatment. As shown in Figure 7C, the 72-hours 5-fluorouracil treatment, there were only minor morphologic changes after 72-hours 5-fluorouracil treatment in Huh-7 pre-S2Δ cells. In contrast, when a combination of 5-fluorouracil with Bcl-2 inhibitor Y II YC137, progressive morphological changes from flat to round in Huh-7 pre-S2Δ cells. Moreover, we examined the effect of 5-fluorouracil or a combination of 5-fluorouracil with Bcl-2 inhibitor II Y137 on modulating the activity of caspase-3 in Huh7 pre-S2Δ cells by using flow cytometric analysis. As shown in Figure 7D, exposure to 5-fluorouracil with or without Bcl-2 inhibitor II Y137 led to stimulation of caspase-3 activity (Fig. 7D). For example, 5-fluorouracil caused 3-fold increase in caspase-3 activity at 0.25 mM. However, when co-incubation of 5-fluorouracil with Bcl-2 inhibitor II Y137, caspase-3 activity was enhanced to 4-fold in Huh-7 pre-S2Δ cells compared with Huh-7 pre-S2Δ control cells (Fig. 7E). In addition, to evaluate early apoptosis and late apoptosis (or necrosis), the effects of 5-fluorouracil or a combination of 5-fluorouracil with Bcl-2 inhibitor II Y137 on Huh-7 pre-S2Δ cells was determined by Annexin V assay (Fig. 7F), which measures the transfer of phosphatidylserine from the inner to the outer membrane of cells and can detect both early and late apoptotic cells. As shown in Figure 7G and H, the early apoptosis and late apoptosis (necrosis) was significantly induced in Huh-7 pre-S2Δ cells after incubation of 0.25 mM 5-fluorouracil with or without Bcl-2 inhibitor for 24, 48, and 72 h. For example, the late apoptosis (necrosis) indices of 5-fluorouracil or a combination 5-fluorouracil with Bcl-2 inhibitor II Y137 in 72 h treatment were 25 and 46%, respectively.

### The combination of 5-fluorouracil and Bcl-2 inhibitor induces the concomitant inhibition of Bcl-2, Bcl-xL and Mcl-1 expression

To clarify the mechanism by which Bcl-2 inhibitor enhanced the antitumor activities of the 5-fluorouracil, we examined the Bcl-2 family expression status in treated Huh-7 pre-S2Δ cells (Fig. 7I). The result indicated that the combination of 5-fluorouracil with Bcl-2 inhibitor reduced expression level of Bcl-2, Bcl-xL and Mcl-1. In addition, we also evaluated the effects of 5-fluorouracil with or without Bcl-2 inhibitor on phosphorylation status of NF-κB, AKT and ERK in Huh-7 pre-S2Δ cells. As shown in Fig. 7J, NF-κB, Akt and ERK phosphorylation did not affect by 5-fluorouracil or combination treatment.

### Hepatitis B Virus Mutant Large Surface Protein Can Induce Bcl-2 in Vivo

**Figure 8:**
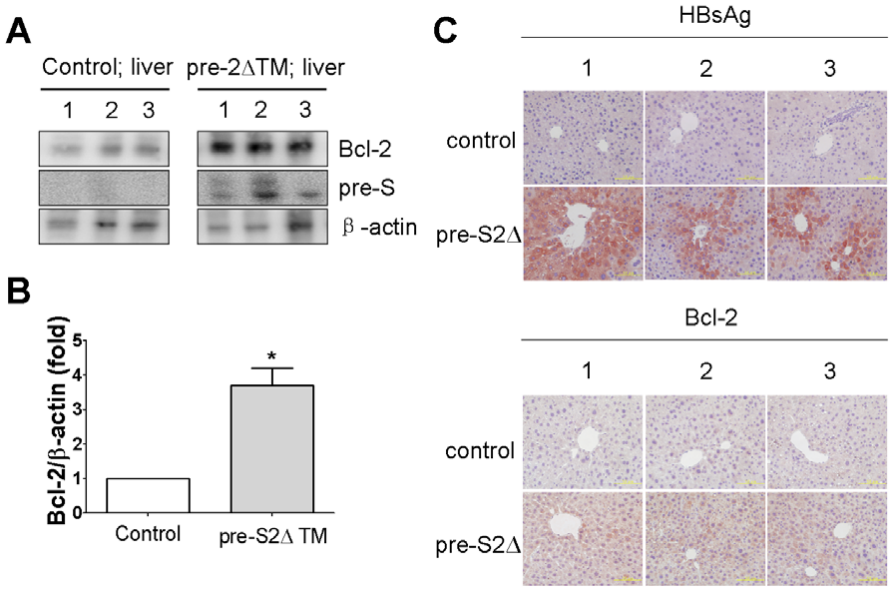
Elevated Bcl-2 is associated with pre-S2Δ expression *in vivo*. (A) Bcl-2 expression was elevated in transgenic mice expressing pre-S2 deletion (Δ2) HBV large surface protein. The expression of Bcl-2 in liver tissues (control and pre-S2Δ) was determined by Western blotting with antibodies for pre-S, Bcl-2, and β-actin for three individual transgenic mice samples. The expression levels of Bcl-2 were quantified by using image J software. The data represent the mean of Bcl-2 protein expression level from three independent experiments. Columns, mean; bars, SD (n = 3). Significant differences (*, *P*<0.05) between the control and experimental group are marked with an asterisks. (C) A marked increase of Bcl-2 expression in pre-S2Δ-expressing hepatocytes was observed. The expression of Bcl-2 and HBV surface proteins in liver tissues of control and pre-S2Δ transgenic mice was analyzed by Immunohistochemical staining (×400).

To confirm further that deletion forms of mutant HBV large surface proteins can induce Bcl-2 *in vivo*, we created transgenic mice that express the pre-S2 deletion form of HBV large surface protein under the control of its native promoter. The expression of HBV large surface protein was detected in the liver (Fig. 8A). Elevated expression of Bcl-2 was observed in liver of pre-S2Δ transgenic mice (Fig. 8B). Furthermore, to examine the event of increased Bcl-2 expression by pre-S2Δ in hepatocytes, both control and pre-S2Δ transgenic mice liver tissues were analyzed by immunohistochemical staining assay. A strong Bcl-2 expression was found in pre-S2Δ transgenic mice hepatocytes. These results altogether demonstrate that Pre-S mutant surface proteins can induce Bcl-2 expression *in vitro* and *in vivo*.

## Discussion

In this study, we show that HBV pre-S2Δ large surface protein enhances *Bcl-2* gene expression and alters chemotherapeutic drug resistance by a mechanism involving *Bcl-2* proto-oncogene expression in human hepatoma cancer cells. Furthermore, expression of Bcl-2 was increased by pre-S2Δ proteins in human immortalized hepatocyte cells and pre-S2Δ transgenic mice liver tissue. In addition, Bcl-2 family, Bcl-xL and Mcl-1, were increased in Huh-7 pre-S2Δ cells. In Huh-7 pre-S2Δ and Huh-7 pre-S cells, ER stress, Akt and ERK phosphorylation were also observed. Moreover, resistant cells caused by expression of HBV pre-S2Δ large surface protein could be sensitized by Bcl-2 inhibitor. Bcl-2 inhibitor overcomes drug resistance to 5-fluorouracil in Huh-7 pre-S2Δ cells, which provides a new approach to the combinational therapy of HBV pre-S2Δ large surface protein-related HCC. Overall, our data provide evidence for relationship between Bcl-2 and HBV pre-S2Δ, and suggest that *Bcl-2* gene is an important determinant of drug-induced apoptosis thereby modulating resistance to chemotherapy in HBV pre-S2Δ- containing cells.

Modulation of apoptosis may influence resistance to chemotherapy, thus affecting the outcome of cancer treatment. Several mechanisms of chemotherapeutic drug resistance in tumors, such as overexpression of the multidrug resistance gene, increased DNA repair and the multidrug resistance-associated protein, are well understood. An alternation of various regulatory genes in tumor cells may also affect cellular sensitivity to chemotherapeutic drug. The changes in genes expression involve a diverse group of gene products, including oncogenes, tumor suppressor genes, cell cycle regulators, transcription factors, DNA repair factors, growth factor receptors, and cell death regulators. The multiple mechanisms of intrinsic drug resistance are not thoroughly understood and may involve the expression of multiple genes during tumor progression. In this study, Bcl-2 expression was enhanced by HBV pre-S2Δ large surface protein and the consequence of Bcl-2 expression was associated with resistance to 5-fluorouracil in hepatoma cells. In addition, the result has shown that phosphorylation of NF-κB p65 was increased in Huh-7 pre-S2Δ and Huh-7 pre-S cell lines. Transcriptional activity of NF-κB is controlled by phosphorylation of p65 at multiple serine residues. Here we show that induction of p65 phosphorylation at Ser276 and Ser311 sites is induced in Huh-7 pre-S2Δ and Huh-7 pre-S cells. In our previous studies, we demonstrated that activation of NF-κB was enhanced by ER stress through PERK-eIF2α signal pathway, pp38 kinase and calcium signaling [21]. Activation of NF-κB regulates more than 100 gene expressions as far as presently known. These genes have been reported to be involved in diverse physiological conditions of cells, such as inflammation and immune response, cell proliferation, cell differentiation, and apoptosis [40]. The induction of NF-κB activation by HBV pre-S2Δ large surface protein may practically provide anti-apoptotic effect on chemotherapeutic treatment. Taken together, the mechanisms of HBV pre-S2Δ large protein in HCC are not fully understood and need further investigation.

In addition, other previous reports have indicated that constitutive NF-κB activation is found in approximately 15–20% of all cancers [41], [42], and NF-κB has been associated with several aspects of tumorigenesis. NF-κB plays an important role in gene expression in cancer development. It inhibited proapoptotic gene expression, induces anti-apoptotic gene expression [43], stimulates cytokine production [44], induces angiogenesis via VEGF, IL-8, PDGF [45], activation of various cell cycle genes [46], and enhances the expression of the multidrug resistance (MDR) protein and mediates chemoresistance of tumor cells [47]. On the other hand, previous studies have indicated that NF-κB p65 mRNA and protein overexpression were detected in 5-fluorouracil-resistant cancer cell lines [48]. The resistant cell lines had higher NF-κB DNA binding and transcriptional activity. Inhibition of NF-κB activity can enhance the cytotoxicity of some chemotherapeutic drugs. In addition, induction ERK and Akt phosphorylation was observed in both Huh-7 pre-S and pre-S2Δ-expressing cells. Taken together, these results may be able to explain that although protein level of Bcl-2 was not altered in Huh-7 pre-S cells, pre-S-expressing cells exhibited minor drug resistant to 5-fluorouracil treatment.

Our previous studies indicate that overexpression of pre-S2Δ large surface proteins have been demonstrated in the induction of endoplasmic reticulum (ER) stress [18], oxidative stress and DNA damage [49], COX-2 expression [21], cyclin A expression [50], degradation of p27Kip1 [51], vascular endothelial growth factor-A [12], interaction with α-acid glucosidase [52], and lipid upregulation [53]. These results suggested that expression of HBV large surface proteins, especially pre-S2Δ mutant, might be important for hepatocarcinogenesis.

Similar to HBV pre-S2Δ large surface protein, Epstein-Barr virus latent membrane protein 1 has been shown to induce Bcl-2 expression [54]. The up-regulating expression of the cellular oncogene *bcl*-2 by EBV virus latent membrane protein 1 protects infected B cells from programmed cell death. Therefore, induction of the cellular oncogene *bcl*-2 expression has been demonstrated in two different types of virus. The induction of the cellular oncogene *bcl*-2 by other virus-encoded proteins warrants further investigation. In addition, recently, accumulating evidence has indicated that altered miRNA level resulted from mutation or aberrant expression is correlated with gene expression and various human cancers development. For example, miR15b and miR-16 were demonstrated to play a role in the development of MDR in gastric cancer cells by targeting the antiapoptotic gene *BCL2* [55]. Alternation of miRNA level by pre-S and pre-S2Δ in hepatoma cells may also provide important role in resistance to chemotherapeutic drugs.

Chemotherapy for the treatment of cancer became a clinical practice more than 50 years ago. Although the chemotherapy has successfully treated many types of tumors, including HCC, HCC are inherently chemotherapy-resistant tumors and are known to overexpress the drug-resistant genes. Thus, there is a clear need for developing effective, life-prolonging therapeutic strategies for the large number of HCC patients with advanced disease. In this study, we reported that induction of cellular oncogene *bcl-2* expression by HBV pre-S2Δ large surface proteins increased drug resistance to 5-fluorouracil treatment and drug resistance can be significantly decreased by co-incubation with Bcl-2 inhibitor. Because the occurrences of HBV pre-S2Δ large surface proteins in HBV cancer patients are about 30% [18]. Therefore, beside to estimate HBV DNA levels in chronic HBV cancer patients, the HBsAg DNA sequence should be analyzed before undergoing systemic chemotherapy. That information of HBV pre-S2Δ large surface protein with HBV chronic infection is an adverse factor for survival and may be associated with a higher incidence of severe hepatitis during chemotherapy. Taken together, these results may provide a novel chemotherapeutic strategy for HBV pre-S2Δ large surface protein tumor cells.
